# Food as both comfort and risk: perspectives on healthy eating among Swedish women with prediabetes

**DOI:** 10.3389/fgwh.2026.1791140

**Published:** 2026-04-21

**Authors:** Åsa Carlsund, Åsa Hörnsten, Fatima Hoosen, Stephen Amoah, Julia Otten

**Affiliations:** 1 Umeå University, Umeå, Sweden; 2 University of Cape Town, Rondebosch, South Africa; 3 University of Bonn, Bonn, Germany

**Keywords:** experiences, lifestyle, prediabetes, qualitative research, Sweden, women

## Abstract

**Background:**

Prediabetes is a condition in which blood glucose levels are elevated but not high enough to meet the diagnostic criteria for diabetes. It often presents without symptoms but indicates a higher risk of developing type 2 diabetes and other health issues. Without lifestyle changes, many individuals with prediabetes develop type 2 diabetes within a few years.

**Objective:**

This study aimed to explore women's experiences of managing diet in daily life following a diagnosis of prediabetes.

**Methods:**

This qualitative study involved interviews with 16 Swedish women aged 59–72 with prediabetes from the SCAPIS cohort, recruited via convenience sampling. Data were collected from May to August 2025 using open-ended questions. The interviews were recorded, transcribed, and analyzed using qualitative content analysis.

**Results:**

Women viewed food as both sustenance and emotional comfort, creating tension between health goals and emotional needs, complicating diet adherence. The analysis highlighted one main theme, “Imposed demands to take responsibility for one's own and others’ healthy eating habits,” and two additional themes, each with three subthemes.

**Conclusions:**

The results highlight that food serves not only as sustenance but also as emotional comfort, creating tension between health goals and emotional needs, which hampers adherence to dietary advice. Women often have different eating patterns than men, emphasizing the need for person-centered, gender-sensitive approaches.

## Background

Prediabetes is a condition in which blood glucose levels are higher than normal but not high enough to be classified as type 2 diabetes (T2D) (1, 2). It is often asymptomatic but indicates a higher risk of developing diabetes and other serious health conditions (3–5). Without lifestyle changes, many individuals with prediabetes are likely to develop T2D within a few years (6, 7). Globally, the prevalence of impaired glucose tolerance (IGT) was 9.1% in 2021 and is projected to rise to 10.0% by 2045 (8). The lifetime risk of developing T2D for a woman with prediabetes is estimated to range between 57.5% and 80.2% (9). Although the risk of death is significantly higher among individuals who progress to T2D, prediabetes itself still poses a high health risk (10).

Globally, gender differences in risk factors for prediabetes have been identified in previous research (2, 11). A systematic review found that hypertension and poor diet quality were more strongly associated with woman, while abdominal obesity, dyslipidemia, smoking, and alcohol consumption were more prominent risk factors among men. General obesity was identified as a significant risk factor for both genders (12). Social and cultural gender roles influence both lifestyle and health behaviors, which may contribute to differences in risk profiles between men and women. For instance, norms regarding body image, eating habits, and physical activity may vary depending on gender and culture, impacting the ability to prevent prediabetes (13, 14). Working conditions also significantly influence prediabetes risk. Schmidt et al. (15) reported that shift work, work-related stress, and low job control can worsen metabolic risk factors. These factors may interact with gender roles, deepening health inequalities (15).

The recommended treatment for prediabetes involves a combination of lifestyle changes, including dietary adjustments (16), weight loss, and regular physical activity (17). A study confirms that lifestyle changes, including dietary adjustments and increased physical activity, can substantially lower T2D risk in high-risk groups. For instance, lifestyle interventions cut the risk of developing T2D by 47% (RR = 0.53, 95% CI: 0.41–0.67) (18). International guidelines from the International Diabetes Federation (IDF) and the American Diabetes Association (ADA) recommend a person-centered approach that focuses on evidence-based dietary patterns, weight management, and metabolic goals (29, 30). The ADA's 2025 guidelines suggest incorporating plant-based proteins and fiber-rich foods while reducing intake of sugar-sweetened beverages, with a focus on sustainability and long-term adherence (30).

Despite updated guidelines, challenges remain in their implementation. Socio-economic factors, cultural preferences, and limited access to healthy foods often make it challenging to adhere to dietary recommendations. Person-centered nutrition strategies and behavioral support are increasingly seen as crucial components of effective diabetes prevention programs (29). Qualitative research also indicates that individuals with prediabetes face a complex mix of emotional reactions, social expectations, and structural barriers when trying to change their diet, emphasizing the need for tailored, context-sensitive counseling (31–33). The aim of this study was to explore women's experiences of managing their diet in daily life following a diagnosis of prediabetes.

## Materials and methods

### Design

This study employed a qualitative approach, presenting findings from group interviews with women living with prediabetes, which were analyzed using qualitative content analysis (19, 20). This study is part of a larger interdisciplinary project, and to ensure gender-specific insights, parallel data collection, analysis, and presentation were carried out for women and men.

### Participants

Women participating in the Swedish CArdioPulmonary bioImage Study (SCAPIS) in Umeå, Sweden, from April 2024 to April 2025, underwent an oral glucose tolerance test. Women with glucose levels between 8.9 and 12.1 mmol/L were classified as having prediabetes; they received written information about the condition and were advised to consult a healthcare professional for follow-up blood glucose testing 1 year later. Women with prediabetes in SCAPIS who had indicated in their informed consent that they could be contacted for future studies were approached. The first 50 Swedish women who met the inclusion criteria were contacted by ÅC via telephone. If a person did not answer the initial call, it was repeated two additional times. Of the 23 women who responded, 16 agreed to participate in a group interview. Four group interviews were performed, each with four participants. Participants’ ages ranged from 59 to 72 years (mean = 65.5). Regarding residence, six participants lived in rural areas and 10 in small cities. In terms of employment status, seven participants were retired and nine were currently employed.

### Data collection

Participants were recruited through purposive sampling, as described above. Before deciding to participate, all women received oral and written information about the study's aims and procedures and provided informed consent. Data collection was conducted in Swedish between May and August 2025. The group discussions lasted between 54 and 61 min and were recorded using Microsoft Teams. To capture contextual details that might otherwise be missed, a semistructured interview guide containing background and open-ended questions was used. The guide included the following questions: “Have any of you tried to change your diet before? What kind of dietary change was it? What are your thoughts on the connection between the food you eat and the risk of developing type 2 diabetes? If you instead got tailored advise, how would you experience that?” The recordings, including non-verbal expressions, were transcribed into text by the first author for subsequent analysis.

### Analysis

The analysis of the interviews followed the qualitative content analysis approach outlined by Graneheim and her research group (19, 20). Initially, the transcribed texts were read multiple times and discussed among the researchers to develop a general understanding. The text was then divided into meaning units as part of the de-contextualization process. These meaning units relevant to the study aim were condensed, i.e., shortened while preserving their core content, and later labelled/coded. During the forthcoming re-contextualization phase, the codes were compared and grouped according to similarities and differences. These codes were first organized into preliminary groups and later organized as subthemes and themes through interpretation and abstraction, during which a main theme gradually emerged from the analysis. The analysis was not linear but rather a dynamic process that involved repeated movement between raw data and interpretative levels.

### Trustworthiness

To ensure trustworthiness, we considered credibility, dependability, and transferability of our method. Qualitative content analysis was chosen for the analysis of interviews because it is a structured approach well suited to describing variations in experiences (19, 20). To accurately describe and categorize the data while maintaining the integrity of participants’ responses, we employed de-contextualization and re-contextualization processes. Credibility was enhanced by including representative quotations from the transcripts, providing direct examples of participants' experiences, and integrating disconfirming cases to reflect the diversity of experiences. Consensus among the co-researchers helped ensure that interpretations aligned with the participants' intended meanings. Descriptions of the study context, settings, participants, and details of data collection and analysis promote transferability. Quoting participants alongside the findings enables readers to evaluate the relevance of the results to other contexts.

### Ethical considerations

The study followed the ethical guidelines outlined in the Declaration of Helsinki and the guidelines of the World Medical Association. Ethical approval was obtained from the Swedish Ethical Review Authority (Dno: 2025-00193-01-69890). Participation was entirely voluntary, and all participants provided written informed consent. Efforts were made to protect their integrity throughout the study. Each consent form was associated with a unique digit assigned to each participant, which was used consistently throughout all stages of the research.

## Results

Our analysis identified one main theme and two themes, each comprising three subthemes, which are presented in Table 1 and described in detail in the text, with illustrative quotations from the original interview transcripts.

**Table 1:** Main theme, themes, and subthemes.

Main theme	Themes	Subthemes
Imposed demands to take responsibility for one's own and others’ healthy eating habits	Caught in lifelong social demands to manage weight balance and healthy eating	Carrying long-term experience in dieting Struggling with emotional eating Striving to develop sustainable eating habits in the family
	Overwhelmed by new conflicting demands	Viewing food as comfort, but also a risk Trying but having doubts about success Balancing daily life despite an imminent threat

### Imposed demands to take responsibility for one's own and others' healthy eating habits

The main theme illustrates how the participating women experienced pressure to take responsibility for both their own and their families' eating habits. Many participants reported a long history of dieting and difficulties with emotional eating, where food served both as a source of comfort and as a potential health risk. They strived to establish sustainable eating habits compatible with everyday life but encountered conflicting demands, uncertainty about their ability to succeed, and an ongoing need to balance their own health risks with everyday responsibilities.

### Caught in lifelong social demands to manage weight balance and healthy eating

The first theme was developed from three subthemes: “Carrying long-term experience in dieting,” “Struggling with emotional eating,” and “Striving to develop sustainable eating habits in the family.”. These are described individually below.

### Carrying long-term experience in dieting

Participants described weight gain as a persistent challenge throughout their lives, often accompanied by feelings of frustration and low self-esteem. Many expressed a sense of emotional burden from repeated attempts to lose weight, which were often met with setbacks. Over time, this contributed to reduced confidence in their ability to manage their weight effectively. Strategies that had previously worked became less effective, leading to uncertainty and concern.

Ageing was also perceived as making weight loss harder, with results taking longer. Support, especially shared efforts like losing weight with a partner, was considered vital for initiating and maintaining lifestyle changes. Professional guidance, such as consultations with a dietitian combined with practical tools, helped some participants re-establish healthier habits. Despite access to resources, progress was slow, and ongoing support was viewed as crucial for sustaining motivation and lasting changes.

“We lost a few pounds together a few years ago, and we were a great support to each other, because it's not easy to do it on your own. The great thing was that we thought about it together and did it together, supporting each other, which led to better well-being for both of us.” (GI3)

### Struggling with emotional eating

Participants described a recurring pattern of initial motivation followed by a decline in interest. The intention to start a lifestyle change was often linked to specific moments, such as the beginning of a new week; however, this motivation often faded quickly. This cycle led to feelings of frustration and a sense of being stuck in old habits. Participants also reported that poor body image and the emotional impact of weight-related struggles were key concerns in their battle. Many participants shared deep feelings of guilt and disappointment, often stemming from lifelong experiences with weight management. These emotions were further intensified by the perceived difficulty of making lasting changes and the ongoing presence of food temptations.

“For instance, when it starts getting dark and gloomy in the fall, I believe there's a tendency to kind of.. You deserve something nice because it's so dark and dull. That's what I think. I believe it's harder to just forget about it completely.” (GI2)

Participants reported that their focus on enjoyment and treating themselves on various occasions, along with the wide variety of tempting food choices, made it difficult to maintain lasting healthy habits. Participants described a pattern of maintaining control during the week but overeating on weekends. They also mentioned an ongoing tension between their desire to eat healthily and the practical constraints of time, energy, and daily routines. Although they generally avoided processed foods, exhaustion and poor planning often led them to rely on quick, simple, less healthy meals.

“Time and money! I might also think about how much it costs. But we have such an incredible range of products, there are so many fun and delicious things to cook, we're tempted all the time.” (GI1)

A participant explained that preparing meals on weekends was a strategy to make weekday cooking easier. Regular meals were considered important for both physical and emotional health, as hunger often led to irritability and impulses to eat unhealthily. Living alone posed challenges, especially in managing portion sizes. One participant struggled with cooking small quantities and often served food directly from the pot, which led to overeating. Efforts to adopt mindful serving practices, like portioning before eating, were hard to maintain. Concerns about food waste, especially among those living alone, also led participants to finishing leftovers rather than discard them.

### Striving to develop sustainable eating habits in the family

There was a shared understanding that dietary change is not a temporary solution but a lifelong commitment. Participants expressed a desire for lasting results and acknowledged the need for consistency, viewing the process as a permanent shift rather than a passing phase. One participant referred to it as “a life change”, highlighting the lasting impact on the entire family. Participants also described a tension between their desire to eat healthier and the realities of everyday life shaped by family routines, cultural food traditions, and practical constraints of time and energy. They found themselves navigating competing demands, balancing nutritional ideals with the preferences of children and partners, managing limited time for meal preparation, and reconciling dietary advice with deeply rooted cultural norms around food.

“Losing weight usually involves quite significant changes, and I think that requires making a big adjustment to your diet and sticking to it over time. But the rest of your life can become quite complicated, especially if you live with someone or participate in a social context, then it becomes difficult. Perhaps we might be making too big changes. I think I usually notice when someone talks about making a dietary change, and then it lasts for two or three months, and then it gets so complicated that it's no longer possible.” (GI4)

Participants appreciated practical tools such as weekly food boxes, which helped them eat healthier, especially in large families. These services offered convenience, reduced the mental and logistical burden, and minimized the risk of unhealthy grocery choices. They enabled women to translate dietary advice into daily actions within family life and made it easier to try new foods aligned with their health goals. Preparing small portions of vegetables was described as a simple strategy to promote healthier eating. Some participants aimed to cut down on rice, pasta, and potatoes, adopting a flexible approach by moderating intake, with potatoes remaining central due to cultural and personal reasons.

“Yes, we grew up with potatoes, so it's like the old, safe, and familiar stuff. There are lots of healthy things you can do with potatoes, so I find it a little hard to understand why you should avoid potatoes.” (GI2)

### Overwhelmed by new conflicting demands

The second theme was developed from three subthemes: “Viewing food as comfort, but also a risk,” “Trying but having doubts about success,” and “Balancing daily life despite an imminent threat.” These are described individually below.

### Viewing food as comfort, but also a risk

Participants described food as a source of emotional comfort, especially during times of stress and exhaustion. One participant mentioned that unpleasant weather or the struggles of everyday life created a sense of entitlement to eat out of despair; for instance, the darkness and boredom during autumn and winter made them feel they deserved “something nice.” Although participants were aware of dietary guidelines, they acknowledged that adhering to them was challenging due to the demands of everyday life. One participant said that she knew how she should eat, but that “so many factors in life” made it hard to follow these guidelines.

“It's difficult to cook good food in small quantities, that's all. It's very, very difficult. And then there's the issue of portions. When you've finished eating and there's a little bit left, you take it out of habit. I almost always do that.” (GI3)

Food was often seen as a reward, especially during times of emotional strain. Even participants who did n't usually crave sweets admitted to weakening their dietary rules when tired or stressed, explaining that they allowed themselves more treats in such situations. Rich flavors were highly appreciated because they enhanced the overall dining experience. One person noted that these ingredients were difficult to give up because they increase the taste of other ingredients.

“What I find hard to do without, and what I believe improves the flavour, is butter and cream.” (GI1)

### Trying but having doubts about success

Participants explained that medical emergencies served as critical moments that prompted immediate lifestyle changes. One woman said that it usually takes a dramatic wake-up call to get started, comparing the experience to “having a knife to your throat.” For her, this sense of urgency was necessary to overcome her low interest and begin adopting healthier habits. As a result, she reported that her laboratory test results had remained stable and positive since changing her diet.

“Now that you know you are actually ill, it becomes more specific, more concrete. Then it becomes clear what you can do to get better. You're not getting any younger, but you have to keep on living.” (GI4)

Receiving specific health information, such as elevated cholesterol or a diagnosis of prediabetes, made the need for change feel more concrete. One participant explained that learning about her prediabetes clarified her steps she needed to improve her health. Motivation extended beyond weight loss to broader health goals. For example, a woman taking cholesterol medications wanted better overall balance in her health rather than focusing just on weight. She was happy with her progress and felt more energetic. Several participants mentioned that they did n't think about their diagnosis daily but valued small, manageable changes over drastic ones. Major transformations were seen as emotionally draining and unmanageable, leading participants to express a desire for healthcare support.

“I think I would like to see (my personal) dietary guidelines before I can say how likely it is that I will be able to adhere to them. I would also like, for example, a collection of recipes and other practical tools, such as apps that can support everyday planning and stress management, so that I don't have to make all the plans myself.” (GI3)

### Balancing daily life despite an imminent threat

For some participants, a diagnosis of prediabetes served as a turning point, increasing awareness and encouraging more deliberate choices about healthier eating and leisure activities. However, others described a complex relationship between their lifestyle habits and their understanding of health risks. They saw it as a constant balance between wanting to live, feel healthy, and avoid being overweight, and the temptation of tasty food and a comfy sofa. Many participants were strongly motivated to prevent their condition from progressing to T2D. Many women believed that lifestyle changes, especially weight loss, could lead to noticeable improvements in metabolic health, which boosted their dedication to healthier routines. Still, there were differences among the women. Some had developed strategies that worked well for them, while others doubted their ability to succeed.

“I'm the kind of person who thinks, yes, on Monday I'll start, then I will, and then it's like before Tuesday I've forgotten.” (GI4)

Family history emerged as a factor influencing perceptions of risk. Several participants had close relatives affected by T2D, cardiovascular disease, or stroke, which contributed to a heightened sense of vulnerability and anxiety. Personal experiences of inactive lifestyles and weight-related health problems among family members further contributed to participants' concerns about hereditary risk factors.

“It's clear that I have a pretty strong genetic predisposition, since my dad has diabetes, as do all three of his siblings. Weight is something I've struggled with my whole life, more or less.” (GI2)

Participants also highlighted a desire for continuous follow-up meetings with healthcareprofessionals to maintain motivation and manage daily life challenges. In addition, they expressed a desire for detailed individual dietary advice that is clearly explained before they commit. One participant stated that practicality and personal relevance are most crucial for accepting individual advice.

## Discussion

The findings reveal a complex and emotionally charged landscape in which women with prediabetes manage their health behaviors. Many described a long-term struggle with weight management, characterized by repeated efforts, emotional setbacks, and shifting challenges over time. In addition, the women's narratives demonstrate that food is not just a nutritional necessity but also a multifaceted symbol of comfort, reward, and coping. This duality, where food serves both as a source of emotional relief and as a perceived health risk, highlights the psychological tension many individuals face when trying to follow dietary guidelines. Documented gender differences in dietary intake highlight the importance of tailoring nutritional interventions to address not only metabolic needs but also emotional and behavioral patterns that vary between men and women (21, 22). This underscores the necessity for gender-sensitive dietary advising that considers both biological and psychosocial aspects of eating behavior (23). Previous research also indicates that men tend to consume more energy, protein, and total fat, while women generally have a higher intake of carbohydrate (24, 25). Studies by Ciarambino et al. (13) and Kautzky-Willer et al. (14) highlight how biological, hormonal, and social factors differ between women and men, influencing the course of the disease. These insights are crucial for providing more accurate and person-centered advice and treatment. Psychosocial factors such as stress, caregiving duties, and socioeconomic challenges can further heighten the risk. These findings are crucial when considering how dietary advice is received and implemented.

Participants viewed food as both comforting and risky, especially under stress and exhaustion. This finding aligns with evidence indicating that emotional eating contributes to weight gain and challenges in losing weight (34). Emotional eating, often triggered by negative emotions, can lead to increased consumption of energy-dense, nutrient-poor foods (35). The cycle of motivation, starting strong but fading, reflects the mental burden associated with repeated dieting and reduced self-efficacy. Konttinen et al. (35) found emotional eating mediates the relationship between depression and long-term weight gain. Emotional eaters also tend to experience body dissatisfaction and feelings of guilt, further hindering the adoption of healthy habits (34).

Emergency medical conditions were described as critical motivators for lifestyle change. These “wake-up calls” prompted participants to reassess their habits and commit to healthier routines. This experience reflects Health and Illness Transition Theory developed by Meleis, which helps explain how people move from a perceived state of health to recognizing a health risk. The theory emphasizes the emotional and behavioral adjustments individuals make during such transitions, as well as the importance of support in navigating them (36). However, participants also highlighted that while these moments sparked motivation, they preferred gradual adjustments over drastic transformations. This approach is supported by behavioral research showing that sustainable habit formation is more effective when changes are introduced gradually (26).

Support from partners and healthcare professionals was considered crucial. Collaborative efforts, such as dieting with a spouse or receiving person-centered dietary advice, were described as motivating and effective. These findings align with intervention studies demonstrating that strong interpersonal contact and behavioral strategies are associated with better health outcomes (27).

Participants expressed a desire to establish sustainable dietary habits within family life, seeing dietary change as a “life change” necessary for long-term health. However, cultural food traditions, children's preferences, and time constraints often posed challenges. A Swedish study identified a metabolic signature linked to healthy eating, which was associated with reduced risks of diabetes and cardiovascular disease (37). This offers an objective measure of dietary adherence and health, overcoming limitations linked to self-reported data. Another Swedish study suggests that following national dietary guidelines can improve metabolic health and the gut microbiome (38).

## Limitations

Our study has several limitations. Data collection faced challenges that may have affected sample representativeness, as recruitment calls in May and data collection in June–August coincided with Swedish summer activities, leading many participants to decline due to seasonal routines. Most participants being Swedish-born limits the diversity of perspectives, reducing broader applicability of findings. The use of Microsoft Teams may also have caused methodological issues, such as difficulties in observing non-verbal cues and technical disruptions, potentially limiting comfort when discussing sensitive topics. The small sample size limits the generalizability of the findings; however, in a qualitative context, individual experiences are valuable. The findings reflect the experiences of this group of Swedish women with prediabetes, not those of broader populations, although the rich data may be transferable to similar settings.

## Conclusion

The results show that women with prediabetes face complex challenges when attempting dietary and lifestyle changes. Food serves not only as sustenance but also as a source of emotional comfort, creating tension between health goals and emotional needs, which can hinder adherence to dietary advice. Biological and social factors, such as hormonal shifts and socioeconomic issues, affect disease development and women's receptiveness to guidance. Women often have different eating patterns than men, highlighting the need for person-centered, gender-sensitive advice. Effective dietary guidance must consider women's circumstances, emotional needs, and cultural contexts. Gradual lifestyle changes, family strategies, and strong support are important for achieving sustainable lifestyle adjustments.

## Data Availability

The raw data supporting the conclusions of this article will be made available by the authors, without undue reservation.
